# Neonatal Cardiac ECMO in 2019 and Beyond

**DOI:** 10.3389/fped.2019.00327

**Published:** 2019-08-21

**Authors:** Peter Paul Roeleveld, Malaika Mendonca

**Affiliations:** ^1^Pediatric Intensive Care, Leiden University Medical Center, Leiden, Netherlands; ^2^Pediatric Intensive Care Unit, Children's Hospital, Inselspital, Bern University Hospital, Bern, Switzerland

**Keywords:** ECMO, neonate, cardiac, heart failure, post-cardiotomy, single ventricle, selection criteria

## Abstract

Worldwide, the use of Extracorporeal Membrane Oxygenation (ECMO) for cardiac failure has been steadily increasing in the neonatal population and has become a widely accepted modality. Especially in centers caring for children with (congenital) heart disease, ECMO is now an essential part of care available for those with severe heart failure as a bridge to recovery, long term mechanical support, or transplantation. Short-term outcomes depend very much on indication. Hospital survival is ~40% for all neonatal cardiac ECMO patients combined. ECMO is being used for pre- and/or post-operative stabilization in neonates with congenital heart disease and in neonates with medical heart disease such as myocarditis, cardiomyopathy or refractory arrhythmias. ECMO use during resuscitation (ECPR) or for sepsis is summarized elsewhere in this special edition of Frontiers in Pediatrics. In this review article, we will discuss the indications for neonatal cardiac ECMO, the difficult process of patients' selection and identifying the right timing to initiate ECMO, as well as outline pros and cons for peripheral vs. central cannulation. We will present predictors of mortality and, very importantly, predictors of survival: what can be done to improve the outcomes for your patients. Furthermore, an overview of current insights regarding supportive care in neonatal cardiac ECMO is given. Additionally, we will address issues specific to neonates with single ventricle physiology on ECMO, for example cannulation strategies and the influence of shunt type (Blalock-Taussig shunt vs. “right ventricle to pulmonary artery” shunt). We will not only focus on short term outcomes, such as hospital survival, but also on the importance of long-term neuro-developmental outcomes, and we will end this review with suggestions for future research.

## Introduction

**Figure 1:**
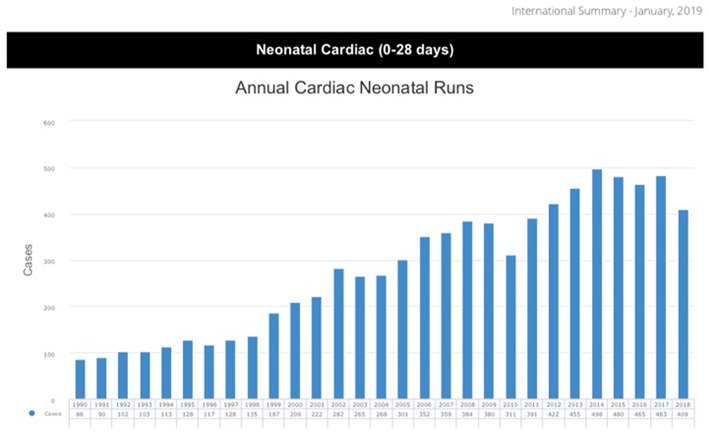
ELSO data report 2019 (1).

Since the inception of Extracorporeal Membrane Oxygenation (ECMO), the number of annually performed ECMO runs for neonates with cardiac disease has been steadily increasing (1) (Figure 1). In the last decade, ~400–500 neonates have been supported with ECMO each year in centers reporting to the Extracorporeal Life Support Organization (ELSO). Since 1987, more than 8,000 neonates have been registered in the ELSO database (1). Hospital survival is ~40% and hasn't really changed since 1987, despite increased experience, better equipment and enforced education and team training. This might be due to a constant widening of indications, increasing levels of complexity and acuity (2, 3).

In hospitals caring for children with heart disease, ECMO has become an essential part of modalities available to temporarily support these neonates when conventional therapy fails. ECMO, however is not a treatment in itself. The goal of ECMO support is to offer the failing myocardium a chance to recover while the body is provided with adequate blood supply. Most often, ECMO for neonatal cardiac disease is used as a bridge to recovery, bridge to long-term support, as a bridge to heart transplantation or bridge to decision making (e.g., diagnostic work-up, organ donor). However, ECMO is no longer a rescue treatment *per se*, as it has also found its way as elective support during diagnostic and therapeutic procedures (4).

In this review article, we will discuss the indications for neonatal cardiac ECMO, the difficult process of patients' selection, identifying the right timing to initiate ECMO, and outline pros and cons for peripheral vs. central cannulation. We will present predictors of mortality and, very importantly, predictors of survival: what can be done to improve the outcomes for your patients. It is essential that explanations are sought for the circulatory compromise leading to ECMO, so attempts can be made to correct possible reversible diseases, such as residual lesions following congenital heart surgery. It is also extremely important to decompress the heart as much as possible to decrease myocardial work and oxygen consumption aiding in recovery. Furthermore, an overview of current insights regarding supportive care in neonatal cardiac ECMO is given. Additionally, we will address issues specific to neonates with single ventricle physiology on ECMO, for example cannulation strategies and the influence of shunt type (Blalock-Taussig shunt vs. “right ventricle to pulmonary artery” shunt). We will not only focus on short-term outcomes, such as hospital survival, but also address the importance of long-term neuro-developmental outcomes and we will end this review with suggestions for future research.

## Indications

Neonates receiving ECMO for cardiac disease constitute a heterogenous group and can receive ECMO for surgical or medical cardiac disease, such as cardiomyopathy, myocarditis, and/or arrhythmias (Table 1).

**Table 1:** Neonatal cardiac runs by diagnosis in the last five years (2014–2019), ELSO registry report January 2019 (1).

	**Total runs**	**Avg run time**	**Longest run time**	**Survived**	**% Survived**
Congenital defect	1,487	144	1,481	698	46%
Cardiac arrest	15	157	600	6	40%
Cardiogenic shock	77	153	1,746	43	55%
Cardiomyopathy	27	231	848	15	55%
Myocarditis	25	250	628	13	52%
Other	621	168	3,737	342	55%

*Avg, average. Run times are given in hours*.

### Congenital Heart Disease

In neonates with surgical cardiac disease, ECMO can be utilized for pre-operative stabilization (e.g., transposition of the great arteries with pulmonary hypertension), failure to wean from cardiopulmonary bypass (CPB), or low cardiac output syndrome (LCOS) post-operatively. Following congenital heart surgery, ECMO is utilized in 1.4–5% of operations (5–7). Cardiac arrest as an indication for ECMO is called Extracorporeal Cardiopulmonary Resuscitation (ECPR) and is discussed elsewhere in this Research Topic of *Frontiers in Pediatrics*.

According to a recent Society of Thoracic Surgeons (STS)-database study, risk factors for receiving post-operative ECMO include young age (13 vs. 195 days), low weight (3.4 vs. 6.4 kg), mechanical ventilation prior to surgery (37 vs. 15% chance of receiving ECMO), arrhythmia (4.6 vs. 2.6%), shock (7.4 vs. 1.7%), higher complexity as indicated by ‘STAT’ category of 4–5 (72 vs. 34%), and CPB-duration (175 vs. 94 min) (5). Also, more complex lesions have a higher chance of receiving ECMO post-operatively. For instance, in neonates following Norwood operations, incidence of ECMO is much higher with ~13% of neonates receiving mechanical support post-operatively (8). Also, higher Vasoactive-inotropic scores have been associated with increased ECMO utilization (9) (Table 2).

**Table 2:** Risk factors for receiving ECMO following neonatal heart surgery.

**Risk factors for receiving post-cardiotomy ECMO**	
Young age	STAT category 4–5
Lower weight	CPB duration
Mechanical ventilation pre-operative	–
Arrhythmia	Shock
Higher vasoactive-inotropic score (VIS)	–

*STAT, Society of Thoracic surgeons—European Association for cardio-Thoracic surgery; CPB, cardiopulmonary bypass*.

### Myocarditis/Cardiomyopathy

The incidence of myocarditis in neonates is not clearly known because the diagnosis remains challenging due to non-specific symptoms, often masquerading as respiratory or gastrointestinal infection. In 2017, a group from Finland analyzed the occurrence and features of childhood Myocarditis and found an incidence of 1.95/100,000 person-years (10). Interestingly, two peaks in occurrence were noted: in infants <1 year and in teenagers. Myocarditis in newborns is mainly caused by viral infection, such as enterovirus, parvovirus, or adenovirus (11).

Looking at the ELSO data registry, out of 2,252 neonatal cardiac runs (2014–2018), myocarditis was listed in only 25 cases as the reason for ECMO initiation (1). A recently published article from Melbourne described a series of seven neonatal myocarditis cases requiring ECMO due to Enterovirus infection (12). In this article, Cortina et al. also included 35 cases of Enterovirus Myocarditis supported with ECMO from literature review in their data analysis. The survival rate of all those cases together was 36% (15/42), which is lower than survival (to discharge or transfer) reported in the ELSO registry with 52% for this population (12).

Extracorporeal Membrane Oxygenation (ECMO) support for neonatal myocarditis is infrequent and still carries a high risk of complications and death, but in some cases can lead to complete cardiac recovery with favorable long-term outcome. But who would benefit from ECMO support and when to initiate it?

Casadonte et al. investigated risk factors for cardiac arrest or mechanical support (MCS) in children with fulminate myocarditis (13). The average age of the 28 patients in this study was 1.2 years (1 day−17 years), but no subgroup of true neonates was analyzed. They found that patients in the CPR/MCS group had higher peak b-type natriuretic peptide (BNP) and peak inotropic score. Unfortunately, there was no marker on admission identified, which could be used as a prediction tool. Other authors found associations between the need for MCS and significant arrhythmias or evidence of end organ dysfunction (14, 15).

Neonates with fulminate myocarditis are at risk for cardiovascular collapse leading to CPR and/or MCS. Unfortunately, no clinical variables predict the probability of MCS or the outcome. In children, ECMO has been shown to improve survival for circulatory collapse due to arrhythmias in myocarditis (16). Further collaboration and research in this field is needed to shed some light on the unknown elements.

A recent meta-analysis of myocarditis and ECMO described a 54–83% long-term survival with optimistic quality of life (17). The analysis included six studies, and all used slightly different criteria for initiation of ECMO, but the authors concluded that a systolic BP < 50 mmHg for neonates despite >2 inotropes or high inotrope score could be a reasonable threshold.

### Arrhythmias

Neonatal arrhythmias can occur post-operatively (e.g., atrial tachycardia, junctional ectopic tachycardia, ventricular tachycardia, or complete heart block), as part of myocarditis/cardiomyopathy as discussed above, or as a primary arrhythmia (e.g., re-entry supraventricular tachycardia or Brugada syndrome). If the arrhythmia leads to refractory shock despite pharmacological treatment, ECMO can be indicated (18–20). During ECMO support, pharmacological treatment can be optimized, cardiac catheterization with possible ablation of accessory pathways can be performed, or a pacemaker can be implanted. For total AV-block, a pacemaker can be placed while supported with ECMO. The utilization of ECMO for arrhythmias is rare but carries very good survival and neurologic outcomes (21).

### Pulmonary Hypertension

The indication of ECMO for persistent pulmonary hypertension of the newborn associated with diaphragmatic hernia, meconium aspiration, respiratory distress syndrome, or sepsis is beyond the scope of this review. In a large database study of neonates and children with pulmonary artery hypertension, 1.4% received ECMO with a hospital mortality of 39% and significant complications (22). ECMO for pulmonary hypertension associated with congenital heart disease has good outcomes (23, 24). Said et al. reported the example of pulmonary hypertension associated with transposition of the great arteries (25). Pulmonary hypertension following cardiac surgery is often due to temporarily increased pulmonary vascular resistance because of cardiopulmonary bypass and probably has better ECMO survival than pulmonary artery hypertension.

## Timing—When to Initiate ECMO?

In neonates following congenital heart surgery, ECMO can be initiated in the operating room for failure to separate from cardiopulmonary bypass or in intensive care for either LCOS resistant to maximal medical therapy or hypoxia. Reports in the literature vary (see Table 3), and unfortunately offer not enough guidance as to the optimal timing of ECMO initiation. Although it might be logical to assume that earlier support may lead to better outcomes, neonates who fail to separate from bypass might be sicker and have worse myocardial depression than those that do separate from CPB and then develop worsening of LCOS although there is no data to support this hypothesis. Earlier reports suggested that failure to separate from CPB was a risk factor for increased mortality (27, 28). However, a recent ELSO database study among more than 4,000 neonates with congenital and acquired heart disease suggests that earlier initiation of ECMO may reduce mortality due to a decreased degree and duration of acidosis prior to ECMO. The authors hypothesized that acidosis probably reflected poor cardiac output and tissue hypoperfusion and that delayed use of ECMO may result in prolonged exposure of the myocardium and end-organs to reduced oxygen delivery resulting in severe or permanent myocardial or end-organ injury and reduced survival (31). But in a recent large retrospective study longer time between surgery and ECMO initiation was not associated with higher mortality although it was associated with longer ECMO duration, prolonged length of ventilation, and prolonged length of ICU and hospital stay (32).

**Table 3:** Hospital survival based on location of ECMO initiation in either operation room (OR) because of failure to wean from bypass or in the intensive care unit (ICU) due to low cardiac output syndrome or hypoxia (7, 26–30).

			**Hospital survival (%)**		
	**Population**	**Overall survival**	**OR**	**ICU**	**Statistical significance**
Jaggers et al. (26)	*N* = 35 (median age 19 days)	60	60	60	NS
Kolovos et al. (27)	*N* = 74 (median age 17 days)	50	64	41	*P* = 0.06
Chaturvedi et al. (28)	*N* = 81 children (median age 2.4 months)	49	64	29	*P* = 0.003
Sasaki et al. (7)	*N* = 36 (median age 64 days)	47	43	60	NS
Casadonte et al. (13)	*N* = 90 (age 6–912 days)	73	77	62	NS
Khorsandi et al. (30)	*N* = 66 Age <16 years	44	47	38	NS

Timing to initiate ECMO therefore remains very difficult. There are no definitive cut-off points and no evidence-based guidelines exist as to when to initiate ECMO post-operatively. The decision to proceed to ECMO cannulation is typically made on a case-to case basis based on the experience and judgment of the multidisciplinary team, which is reflected by a substantial variation in the use of mechanical support across hospitals (5, 30, 32). The ELSO advises to consider ECMO for patients with evidence of inadequate end organ perfusion and oxygen delivery resulting from inadequate systemic cardiac output (see Table 4) (33).

**Table 4:** Indications to cardiac ECMO according to the ELSO guidelines (33).

**Use of extracorporeal life support for cardiac failure should be considered for patients with evidence of inadequate end organ perfusion and oxygen delivery resulting from inadequate systemic cardiac output**
(a) Hypotension despite maximum doses of two inotropic or vasopressor medications.
(b) Low cardiac output with evidence of end organ malperfusion despite medical support as described above: persistent oliguria, diminished peripheral pulses.
(c) Low cardiac output with mixed venous, or superior caval central venous (for single ventricle patients) oxygen saturation <50% despite maximal medical support.
(d) Low cardiac output with persistent lactate >4.0 mmol/l and persistent upward trend despite optimization of volume status and maximal medical management.

## Contraindications

The number of contraindications has decreased over recent years as experience and technology have advanced. The most important contraindication is lack of possible myocardial recovery and/or contraindications to heart transplantation. The ELSO guidelines list absolute and relative contraindications (Table 5). The absolute contraindications have an inappropriate chance of major complications and poor outcome, and therefore ECMO should not be considered in those patients. The relative contraindications also carry a high risk of poor prognosis, but careful management may lead to acceptable outcomes. But parents and the medical team involved should all be aware of the high stakes and be prepared to withdraw ECMO if irreversible damage should develop.

**Table 5:** Contraindications to cardiac ECMO according to the ELSO guidelines (33).

**Use of ECLS is not recommended under certain circumstances, particularly if there is strong evidence for lack of capacity to recover or be treated**
**1. Cardiopulmonary extracorporeal life support is inappropriate if**
(a) The condition is irreversible and/or,
(b) There is no timely, reasonable therapeutic option and/or,
(c) High likelihood of poor neurological outcome.
**2. Absolute contraindications: Extracorporeal life support is not recommended in the following circumstances**
(a) Extremes of prematurity or low birth weight (<30 weeks gestational age or <1 kg)
(b) Lethal chromosomal abnormalities (e.g., Trisomy 13 or 18)
(c) Uncontrollable hemorrhage
(d) Irreversible brain damage
**3. Relative contraindications**
(a) Intracranial hemorrhage
(b) Less extreme prematurity or low birth weight in neonates (<34 week gestational age or <2.0 kg)
(c) Irreversible organ failure in a patient ineligible for transplantation
(d) Prolonged intubation and mechanical ventilation (>2 week) prior to ECLS

## Peripheral Vs. Central Cannulation

Due to the size of the neonatal patients, cannulation for cardiac neonatal VA-ECMO can be done two ways:

1. Peripheral cannulation: drainage via jugular vein and return via the carotid artery. The right side is the side of choice, but the left side is also possible.
2. Central cannulation: with chest opening and drainage directly out of the right atrium and return into the aortic arch.

Regardless which way is selected, the adequate size of the cannulas (drainage and return) is crucial to be able to achieve the desired ECMO flow (100–150 ml/kg/min or 3 l/m^2^/min). Every cannula has a pressure/flow chart which describes its characteristics, so the selection is easily facilitated. Most often, the largest possible cannula is inserted.

Cannulation via right carotid artery provides very good hemodynamic support, with flow to the upper body and the descending aorta, though blood flow of the right cerebral hemisphere depends on an unhindered circle of Willis. Neurological complications (such as intracranial hemorrhage, derangement of cerebral autoregulation, impairment of venous drainage and risk of embolic events) are well-described in this population (34, 35).

Other problems reported with this type of peripheral cannulation approach are dissection of the aorta or carotid artery (36). Early recognition and timely intervention to those complications are critical, and serial Echocardiograms should be provided to detect problems. For further clarification CT angiogram or catheter investigations can be helpful to identify the problem.

In some cases, the desired flow cannot be achieved via the neck cannulation, so the central route is chosen, and for inability to wean from cardiopulmonary bypass, the simple conversion to central ECMO is obvious (37). When converting CPB to ECMO, one should be aware that the arterial CPB cannula might not be large enough to support a normothermic patient for several days, especially if sepsis should develop. Complications of central cannulation include bleeding, vessel injury and embolic phenomena. A recent case report alluded to the occurrence of thrombus in the aorta (38). The cannula inserted in the aorta increases the afterload of the ventricle. Thrombosis can occur as a result of stasis within the aorta due to competing flows from the poorly ejecting native ventricle and the ECLS circuit.

Overall cannulation approaches for neonatal VA ECMO have not changed over decades, and unfortunately complications rates remain the same (1).

## Predictors of Survival

Survival depends very much on the underlying reason for receiving ECMO support. Overall survival is higher in non-surgical heart disease such as myocarditis or cardiomyopathy (see Table 1). In neonates with congenital lesions, the risk factors of requiring ECMO post-operatively are listed in Table 2 and are especially high following neonatal Ross-Konno repair [Odds Ratio (OR) 70], Truncus arteriosus repair (OR 42), arterial switch operation with VSD (OR 35), ALCPA-repair (OR 20), TAPVD repair (OR 18), or Norwood operation (OR 9) (5). And subsequently, mortality during ECMO is also very dependent on underlying diagnosis and type of operation. According to the STS database, mortality following Ross-Konno repair or Truncus repair is ~70%, whereas mortality for ECMO following ALCPA repair is only 14% (5). In neonates with ECMO for congenital heart disease reported to ELSO, survival is between 40 and 51% (see Table 6).

**Table 6:** Hospital survival of neonatal cardiac ECMO by congenital diagnosis.

**Congenital lesion**	**Number of runs**	**Survival (%)**
Left to right shunt	92	45
Left-sided obstructive lesion	87	47
Hypoplastic left heart syndrome	439	43
Right-sided obstructive lesion	52	40
Cyanotic—increased Qp	73	43
Cyanotic—pulmonary congestion	167	47
Cyanotic—decreased Qp	295	50
Other	282	51

*Qp, pulmonary blood flow (1)*.

Many outcome predictors have been identified in the literature and are presented in Table 7 (7, 8, 23, 31, 39–45). Some predictors cannot be modified before or during ECMO, such as age, weight, the presence of chromosomal abnormalities, or the underlying diagnoses. Other predictors are determined by the pre-ECMO clinical course, such as inotrope score, duration of ventilation, presence of fluid overload, and CPR requirement. These predictors could possibly be influenced by early timing of ECMO initiation before these predictors occur.

**Table 7:** Predictors of mortality and survival of neonatal and pediatric cardiac ECMO (7, 8, 23, 31, 39–45).

**Predictors of mortality**	
**Higher mortality**	**Lower mortality**
Younger age	
Low bodyweight (<3 kg)	Bodyweight >3.3 kg
Chromosomal abnormalities	No chromosomal abnormalities
Congenital heart disease	Myocarditis/cardiomyopathy
Single-ventricle physiology	Two-ventricles
High inotrope score	Low inotrope score
Duration of ventilation pre-ECMO >14 days	Duration of ventilation pre-ECMO <14 days
CPR pre-ECMO	No CPR pre-ECMO
Acidosis pre-ECMO (pH <7.26)	No acidosis (pH > 7.28)
High Lactate pre-ECMO	Low lactate pre-ECMO
Failure to clear lactate <24 h	Able to clear lactate <24 h
Renal failure	No renal failure
Fluid overload on ECMO initiation	No fluid overload
Organ system complications	No organ system complications
Bleeding during ECMO	No bleeding
Cardiac catheterization on ECMO <48 h	Late or no cardiac catheterization
Duration of ECMO support >7 days	Duration of ECMO support <5 days

Risk factors for poor outcome that may be modifiable during ECMO support include the identification of residual lesions, optimizing systemic perfusion (reflected for instance by clearance of acidosis), presence of renal failure, and fluid overload, and the duration of ECMO. As mentioned before, it is essential to determine the reason the patient requires ECMO support, and to provide optimal systemic perfusion while resting the heart as much as possible and limiting the duration of ECMO support and concomitant complications which can impact outcomes.

### Residual Lesions

Recently, several studies have shown the importance of early catheterization aimed at identifying and treating residual lesions (43, 44, 46, 47). In a retrospective study by Agarwal et al., residual lesions were present in approximately one-quarter of post-operative cardiac surgery patients receiving ECMO support (43). They conclude that all post-operative pediatric cardiac surgery patients unable to be weaned off ECMO successfully, should be evaluated actively for residual lesions, preferably by cardiac catheterization, as echocardiography only detected 20% of all residual lesions and catheterization the remaining 80%. Furthermore, earlier detection (within 3 days of ECMO support) and reintervention are associated with improved clinical outcome. In another retrospective study of 84 neonates requiring ECMO following cardiac surgery, as many as 83% had residual lesions (44). Time to identification and/or correction of these residual lesions was significantly shorter in survivors than in non-survivors (1 vs. 2 days). Abraham et al. catheterized 35 neonates while on ECMO support, which led to direct intervention in ~75%, significantly improving survival (46). The average interval from ECMO cannulation to catheterization was significantly shorter in survivors (1.6 days) vs. non-survivors (3.5 days). In a retrospective study by Kato et al., patients who received cardiac catheterization within 48 h after ECMO initiation demonstrated significantly better survival than those who underwent later catheterization (47).

Results of these studies clearly indicate that an early (<24 h) and proactive search for residual lesions is warranted to improve survival. Cardiac catheterization can be safely performed on patients supported by ECMO, and is a critical tool in the early recognition, diagnosis, and direct treatment of hemodynamic and/or anatomic abnormalities (48).

### Clearance of Acidosis

Providing adequate systemic blood flow is an integral aspect of ECMO support. If ECMO flow is inadequate, lactate will remain high as a sign of end-organ hypoperfusion and multiple organ failure may develop impacting on outcomes (44). Therefore, aiming to normalize lactate levels as soon as possible (12–24 h post-cannulation) may improve outcomes. Lactate which has not normalized within 72 h has been associated with decreased survival (44).

### Renal Failure and Fluid Overload

Renal failure and fluid overload at ECMO initiation have been identified as risk factors for poor outcomes in multiple studies (49). Acute kidney injury is probably a reflection of pre-ECMO injury but could also be due to insufficient ECMO flow. Fluid overload, with or without renal failure, may impact on respiratory mechanics and myocardial recovery (49, 50). Renal replacement therapy during ECMO is therefore advocated by ELSO, and has been shown to improve fluid balance and electrolytes (51–54). Renal replacement therapy however does not seem to shorten ECMO duration or ICU length of stay, nor to improve survival (51). It is probably best to prevent the development of renal failure and/or fluid overload prior to initiating ECMO rather than attempting fluid removal while on ECMO.

### Unloading the Ventricle

The goal of cardiac ECMO is to rest the myocardium as much as possible so it may recover as soon as possible. However, by increasing the afterload of the systemic ventricle by placing an arterial ECMO cannula in the aorta or carotid artery, the already failing myocardium may struggle to eject blood against this increased afterload, and a cardiac stun may occur. The aortic valve remains closed and the left ventricle dilates, because on ECMO there is always blood returning to the left atrium from Thebesian and bronchial veins. While dilated, the myocardium stretches and is under strain, therefore diminishing the coronary perfusion and further impacting on myocardial recovery. Also, intracavitary thrombus formation and pulmonary oedema will occur when left atrial pressure exceeds 25–30 mm Hg. Echocardiography is essential in assessing LV distension and presence of spontaneous contrast (55). To provide optimal myocardial recovery, attempts should be made to prevent dilatation of the left side of the heart. The right-side is decompressed by the ECMO circuit. And in lesions affecting RV dysfunction such as pulmonary hypertension or following pulmonary atresia repair, unloading the left ventricle is probably not necessary. In some neonates with LV dysfunction, it may be enough to support the systemic ventricle with a low dose inotrope to open the aortic valve and eject just enough blood to prevent or treat ventricular dilatation. However, in most neonates, this course of action will not be sufficient, and other steps will have to be made. A solution is to unload the left side of the heart by either creating an atrial shunt via percutaneous atrial septostomy or surgical atrial septectomy, or by placing an extra ECMO cannula in the left atrium which drains blood to the inlet side of the ECMO circuit (the so called “left vent”). In neonates, it is not known which form of ventricular unloading is preferred, or what the best timing of unloading is. Currently, there are no standardized diagnostic criteria or guidelines for the type and timing of intervention for LV overload (56). Tentatively, left ventricular unloading should be performed with significant LV distension or spontaneous contrast on echocardiography, when the aortic valve does not open, or with signs of pulmonary oedema on chest x-ray (55, 57).

Elective decompression has been shown to decrease ECMO duration in pediatric cardiac ECMO patients, but not mortality (58). In a single center study from Boston, percutaneous atrial septostomy was used in 10% of all ECMO patients and in 50% of myocarditis patients (57). Residual atrial septal defects requiring closure occurred in only a few cases.

### ECMO Duration

Duration of ECMO support is another important factor and has been associated with decreased survival in several studies (8). With longer ECMO duration, more complications such as renal failure, bleeding, thrombosis, or infection may occur which impact survival. Prolonged ECMO in children with cardiac disease carries much lower survival rates. An ELSO registry study showed that overall ECMO survival is 45% in children with cardiac disease, but drops down to 23–25% survival for ECMO between 14 and 28 days, and 13% for ECMO runs longer than 28 days (40).

## Supportive Care

The Extracorporeal Life Support Organization (ELSO) regularly publishes informative and updated guidelines on pediatric and neonatal cardiac ECMO including supportive care (33). Anticoagulation during neonatal cardiac ECMO is essential and can be very challenging, although it is beyond the scope of this review. In this review, we restrict ourselves to a few important issues specific to neonatal cardiac ECMO. For more information on supportive care (e.g., infections, temperature management, analgesia, and sedation), we refer to the ELSO guidelines and ELSO “Red book” (33, 59).

### Echocardiography

The role of echocardiography in cardiac ECMO is essential in assessing ECMO initiation and separation readiness, cannula positions, and the development of complications such as ventricular dilatation or cardiac tamponade, but also has important limitations as physiological changes induced by ECMO may alter echocardiographic findings (55). An echocardiography-trained physician should be part of the team caring for neonates and children on ECMO, and the use of specific and consistent echocardiographic protocols for patients on ECMO is recommended (55).

### Mechanical Ventilation

Generally, neonates who receive cardiac ECMO have healthy lungs and have not received high ventilation pressures for prolonged periods of time prior to ECMO initiation. Also, relatively short ECMO duration of 5–7 days is expected for these patients. The goal of mechanical ventilation during ECMO is to minimize lung injury and to optimize lung function in order to allow separation from ECMO once myocardial recovery has occurred (33). Therefore, most often, low ventilation pressures can be used during ECMO aimed at maintaining normal tidal volumes (4–6 ml/kg) and preventing alveolar collapse by using PEEP of 8–10 cm H_2_O. No single ventilation strategy is universally practiced, and the suggested target may be inappropriate in patients with an open sternum, poor lung compliance, pulmonary hemorrhage, or intrathoracic hematoma (33). Some neonates, however, may have atelectasis which may require inhalation therapy, physical therapy, and/or bronchoscopy as it is essential to keep the lung open. Therapy resistant atelectasis can be due to tracheal and/or bronchial compression by cardiac or vascular structures which might require contrast CT-scanning to identify.

In neonates with an open sternum, lower ventilator pressures will often suffice unless there is blood or fluid in the thoracic space which should be considered for removal or drainage. Cardiopulmonary bypass and its extension to ECMO have been shown to cause a decrease in lung compliance through reduction in the surfactant activity and surfactant has recently been used safely and effectively in neonatal cardiac ECMO patients with decreased lung compliance (60).

## Single Ventricle Lesions

Extracorporeal support in neonatal patients with single-ventricle physiology is particularly challenging, but also represents a growing and substantial group of neonates who are being supported by ECMO worldwide. Following the stage-one operation for hypoplastic left heart syndrome, 13–20% of neonates will receive ECMO support (8, 61, 62).

General risk factors for requiring ECMO post-stage-one surgery are similar to other post-cardiotomy risk factors, such as low birthweight and longer CPB-time. More specific risk factors include a small ascending aorta (<2 mm), mitral stenosis with aortic atresia, intraoperative shunt revision, and a right ventricular to pulmonary artery shunt (RVPAs) when compared with a modified Blalock-Taussig shunt (mBTs) (62, 63). In the single-ventricle reconstruction (SVR) trial, most neonates who received ECMO post-stage one (70%) failed to separate from CPB and had significantly lower transplant-free survival rates compared to neonates not receiving ECMO post-Norwood (62). In single-center study by Hoskote et al., 56% of post-operative single-ventricle ECMO patients received ECMO because of cardiac arrest, and 44% because of LCOS, with 44% survival to discharge (64). The indication for initiation of ECMO also significantly impacts survival. In a single-center study in 44 neonates, patients cannulated for hypoxemia, and particularly shunt thrombosis, had markedly improved survival (72%) compared to those supported primarily for low cardiac output (21%) (65).

**Figure 2:**
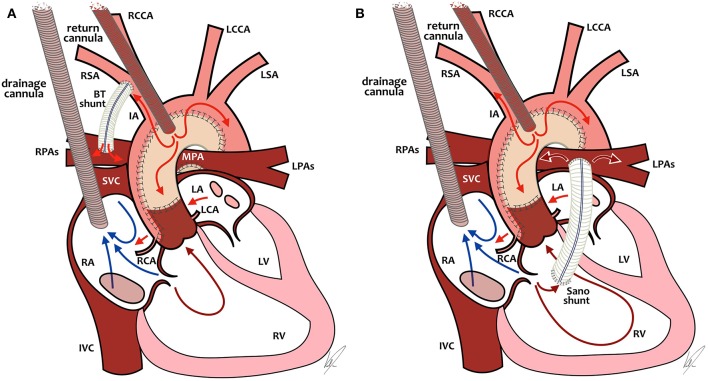
ECMO cannulation and preferential ECMO flows in single-ventricle patients with BT-shunt (**A**, left) or Sano-shunt (**B**, right). In both diagrams **(A, B)**, the arterial cannula is placed in the neoaorta, but it can also be placed through the carotid artery or in the innominate artery. RCCA, right common carotid artery; LCCA, left common carotid artery; LSA, left subclavian artery; RSA, right subclavian artery; IA, innominate artery; RPAs, right pulmonary arteries; LPAs, left pulmonary arteries; MPA, main pulmonary artery; SVC, superior vena cava; IVC, inferior vena cava; RA, right atrium; LA, left atrium; RCA, right coronary artery; LCA, left coronary artery; RV, right ventricle; LV, left ventricle. Drawings by Marta Velia Antonini.

Without going into the complete debate about mBTs vs. RVPAs, which is out of the scope of this review, there is an important difference between the two shunts regarding ECMO management. In the SVR trial, after adjusting for surgeon and birth weight, neonates with a mBTs had smaller chances of receiving ECMO and had significantly better outcome after ECPR or ECMO compared to neonates with a RVPAs (62). Let's take a closer look at ECMO management in those two specific physiologies (Figure 2).

### ECMO and mBTs

Because of the position of the arterial cannula relative to the mBTs and the lower vascular resistance in the pulmonary circulation relative to the systemic circulation, a large proportion of ECMO blood flow will be directed toward the pulmonary circulation, leading to underperfusion of the systemic circulation. In the past, attempts were often undertaken to partially or completely close the mBTs while on ECMO to improve systemic blood flow. That approach of “clipping the shunt,” however, has been shown to increase mortality due to obstruction in the shunt or in the pulmonary vasculature after decannulation and has largely been abandoned in favor of increasing ECMO flow to desirable systemic blood flow, while accepting a significant amount of pulmonary blood flow (26, 66). We therefore recommend a higher blood flow (up to 200 ml/kg/min).

### ECMO and RVPAs

In neonates with an RVPAs, the arterial ECMO cannula is positioned after the source of pulmonary blood flow. This means that if there is no pulsatility of the heart itself, there will be no pulmonary blood flow at all, which leads to the risk of clotting the shunt and/or the pulmonary vasculature. In neonates with some degree of pulsatility, there will be pulmonary blood flow which is dependent on pre-load of the right ventricle (RV) and pulmonary vascular resistance. In that scenario, increasing ECMO flows will lead to decreased pre-load as the drainage cannula will empty the RV and decrease pulmonary blood flow, with the possible risk of thrombosis of the shunt or even the RV. And absent pulmonary flow might lead to increased mortality similar to mBTs patients with clipped shunts (26). All in all, in neonates with an RVPAs, we recommend not to use high flows as in mBTS patients, but to titrate the flow as low as possible to achieve adequate systemic blood flow and also some degree of pulmonary blood flow over the RVPAs. It might be necessary to use some inotropes to promote contractility in this specific group of patients.

Survival to discharge in hypoplastic left heart patients supported by ECMO was 31% as shown in an ELSO registry study by Sherwin et al. (67). Predictors of mortality were pre-ECMO ventilation > 5 days (OR 1.9), pre-ECMO PEEP > 8 (OR 1.9), and increased ECMO duration. In a separate single-center study, survival was 62% and failure to clear lactate within 24 h was also a significant predictor of mortality (45).

A specific group are patients post-hybrid palliation of HLHS. In an ELSO registry study, survival to discharge was only 16% in neonates who received ECMO following stage 1 hybrid palliation (68). This could be because, in many centers, hybrid palliation is reserved for high risk patients, but also could be due to cannulation challenges, and risk of stent compression or even occlusion.

Of concern is late attrition following ECMO in SV survivors. Although incidence is low, mortality is very high (69). Neonates who survive to discharge following ECMO post-Norwood have been shown to have an increased risk of death or cardiac transplant, when compared to patients who did not receive ECMO post-Norwood (45, 61, 63, 70).

For respiratory failure in SV patients, VV ECMO can also be considered as an option with good outcomes (71, 72). In an ELSO registry study of 89 patients with single-ventricle physiology and a median age of 66 days overall survival was 51% for respiratory indications which included neonates as young as 9 days (71). The most common cannulation approach for patients who were unrepaired or palliated with a central or Sano shunt was a double-lumen venous cannula in the right internal jugular vein.

Advantages could be preservation of pulsatile flow and decreased afterload on the heart, as well as avoiding cannulation of the carotid artery which precludes potential run-off through a systemic-to-pulmonary shunt. For more in-depth information on cannulation strategies, timing, circuit flow, and lung rest strategies we refer you to an excellent review by Nair and Oishi from 2016 (72).

## Weaning

In adults, end-tidal CO_2_ (etCO_2_) has been shown to be a useful continuous parameter for predicting the adequate timing of weaning of ECMO for circulatory failure at the bedside (73). Many physicians dealing with neonates also use etCO_2_ as it can indicate increasing pulmonary blood flow as a sign of increased intrinsic right ventricular output due to a healing myocardium or decreasing pulmonary vascular resistance. There is however no known cut-off etCO_2_ when a neonate might successfully separate from ECMO, therefore it can merely be used as an indirect indication of ongoing recovery.

When weaning is not successful and additional time does not lead to adequate recovery, it is important to consider possible transplantation or withdrawal of support and shift attention to comfort of the neonate and guidance for the parents.

Survival to decannulation in neonatal cardiac ECMO is 71%, but drops down to 49% when it comes to hospital discharge or transfer (1). Often, ECMO support is removed during a still fragile state of the patient's recovery. Timing and manner of weaning support highly affect the chances of survival. Unfortunately, up to this date, very little evidence has been published to help identifying parameters which predict readiness to be separated from ECMO. Recommendations about the speed of weaning or acceptable amount of inotropic support are also lacking.

The reason of myocardial dysfunction is a relevant factor for the expected time to recovery and removal of ECMO. Recovery from cardiac dysfunction post-cardiac surgery is expected to happen between 48 and 72 h after initiation of ECMO. The absence of signs of recovery, such as increasing pulse pressure or increasing end-tidal pCO_2_ after that period, should lead instantly to further assessment of remaining cardiac lesions if not already done so (44).

Recovery from a primary myocardial dysfunction, as with myocarditis, occurs over weeks or months, in some cases not at all. Transition to a VAD device as bridge to recovery or bridge to transplant need to be taken into consideration if no evidence of myocardial recovery has occurred within 2 weeks (74).

Weaning can be started with signs of myocardial recovery and adequate resolution of systemic inflammatory response or pulmonary problems (75).

Cardiac function assessment by echocardiography during full ECMO flow does not predict performance of the heart under the completely different circumstances occurring after decannulation (which will be increased preload and decreased afterload) and might only be used as a trend.

Echocardiography under low flow conditions has been shown as being predictive of successful decannulation in adult patients with cardiogenic shock (76).

However, achieving actual low flow conditions in neonates is not possible due to the required minimal flows of the ECMO devices (100–200 ml/min). Therefore, different techniques of weaning trial in which the readiness for separation from ECMO can be assessed (pulse pressure, blood pressure, inotropic needs, echocardiography) are practiced in this age group. In patients with a left-sided drainage cannula, this must be clamped and/or removed allowing restoration of LV pre-load before assessing weaning readiness.

A common approach of weaning ECMO flows in neonates includes inserting a connection (“Bridge”) between the arterial and venous limb (arterio-venous bridge) of the ECMO circuit. This allows to clamp access to the patient, while there remains continuation of flow in the ECMO circuit. The patient and the circuit are isolated from each other. Now the hemodynamics such as blood pressure, CVP, lactate, as well as the demand of inotropic support to remain off circuit, can be assessed and an echocardiography should be performed. This method introduces areas of stagnant blood in stop cocks and the cannulas, with the subsequent risk of clot formation in the ECMO circuit. Most centers flush the cannulas every 10 min and limit this kind of trial off period to around 2 h (59).

Other groups promote trial off with retrograde pump flow of the ECMO circuit (77, 78). During this approach, revolutions per minute (RPM) are lowered until the patient's arterial blood pressure is slightly higher than the post-oxygenator pressure, which results in reversal of the flow. The ECMO circuit becomes an arterio-venous shunt and the patient does not receive circulatory or respiratory support. The advantage of this method is that it has no stagnant blood flow in the cannula and that it places an additional burden on the cardiac output, which therefore reassures of sufficient myocardial function. Not observed or investigated by the groups who practice this approach is the theoretical risk of flushing debris and clots absorbed in the oxygenator with the retrograde flow back into the patient. Further research must be established to further evaluate the risk.

Once readiness to be separated from ECMO is established, the process of decannulation can take place. This includes the optimization of conditions with low dose inotropic support started early enough to reach the patient, lung recruitment, correction of metabolic abnormalities, and attaching pacing wires to the pacemaker ensuring proper function of them. Furthermore, each patient should have a defined plan in place in the event of clinical deterioration after decannulation and weather reinstitution of ECMO is an option.

Decannulation for neonatal cardiac ECMO is done surgically and can be performed either in the ICU or in the operating room. Neck cannulation often leads to vessel reconstruction and the cannulation sites must be investigated carefully for any damage, which might need extended repair, such as carotid artery dissection (36). Furthermore, Di Gennaro et al. published a study showing increased risk of stroke in patients with carotid cannulation (79).

Weaning and decannulation of patients with systemic to pulmonary shunts, which are partially or completely clamped is different. In those cases, readiness to wean must be assessed without decreasing flows as this would lead to desaturations.

Separating from ECMO is a complex process and requires careful assessment and planning from the time of ECMO initiation.

## Neonatal ECMO Circuit Considerations

Circuit technology has greatly advanced over the years, but neonates do deserve some special considerations which are nicely reviewed by Connelly and Blinman (80). Nowadays, most centers have moved from roller pumps to modern centrifugal pumps (81). Detailed discussion of the ECMO circuit is beyond the scope of this review but there are some important issues to consider. First, the relatively large priming volumes for neonates can introduce fluid shifts and can have a pharmacological impact. Second, the relatively low blood flows can make neonates more prone to (circuit) thrombosis and more difficult to wean. And third, in the small neonate accurate cannula position can be more precarious than in larger children or adults, especially in veno-venous double-lumen cannulas.

## Mid and Longterm Outcomes

Neonates surviving cardiac ECMO remain at risk of ongoing health problems, unplanned cardiac interventions, unplanned rehospitalization, neurodevelopmental problems, lower mental scores, language acquisition delays, behavioral problems, and diminished quality of life compared to healthy children, children with chronic conditions, and children with congenital heart disease who did not receive ECMO (82–86).

To gain better insight in these mid and long-term outcomes, protocolized follow-up is important, with the aim of identifying neurodevelopmental delay. Hopefully, by focusing on the future development of these patients and sharing outcomes and interventions through research, the ECMO community can develop interventions aimed at minimizing ongoing health issues and optimizing quality of life for patients and their families after hospital discharge.

## Future (Research)

In neonatal cardiac ECMO, one must face many challenges which are common to almost all neonatal and pediatric ECMO patients, such as infection control and anticoagulation. Exciting developments are being made regarding ECMO anticoagulation, in which attempts are being made to anticoagulate the circuit (rather than the patient) by using nitric-oxide donors in the ECMO tubing (87). The balance between bleeding of the neonate and thrombosis of the circuit remains very delicate, especially in neonates with still developing hemostasis in whom the correlation of coagulation tests with the level of anticoagulant and clinical outcomes remains poor (88).

Other important steps that need to be achieved are related to identifying the right patient and the right time for ECMO. Attempts are being made to develop prediction scores, but, at the moment, these remain restricted to pulmonary ECMO and adult cardiac ECMO (89–92)^1^. No prediction scores exist as of yet for neonatal cardiac ECMO. Hopefully, by gathering more data from large databases such as ELSO or the STS-database, and by performing large multicenter trials, prediction scores can be developed in the future. However, due to the relatively small number of neonatal ECMO patients and many aggregating factors, this will be a challenging task, and identifying “the perfect ECMO candidate” will probably remain a local team decision based on experience and published results of others.

Pharmacodynamic and distribution studies are also needed in this very special group of patients. Neonates with their immature organ function, fast changes in maturation (ontogeny) and significantly increased distribution volume on ECMO, plus unknown absorption in the circuit components, are extremely challenging to manage for adequate medication levels. As mentioned before, research directed on long-term outcomes and quality of life are essential. Not only will they help at early patient intervention, but they can also teach the ECMO community the limitations of ECMO support and will help in deciding to which patients we should offer ECMO and to which patients we should not.

## Conclusions

Extracorporeal Membrane Oxygenation (ECMO) is an invaluable tool for neonates with therapy resistant circulatory failure. Patient selection and timing of ECMO initiation however remain very difficult and is not yet evidence based. Mortality is very much dependent on underlying diagnosis, the ability to provide adequate systemic blood flow, duration of ECMO support and concomitant adverse events and complications. Therefore, after ECMO initiation, attempts should be made as soon as possible to identify concomitant problems whose solution would improve the outcome, such as residual lesions following cardiac surgery or arrhythmias. By adequately addressing those underlying issues and limiting the time on ECMO, preferably <7 days, while resting the heart as much as possible, mortality can be reduced. In non-surgical heart disease, restoration of adequate myocardial function can take longer, sometimes requiring ECMO support up to 2 or 3 weeks.

## Author Contributions

All authors listed have made a substantial, direct and intellectual contribution to the work, and approved it for publication.

### Conflict of Interest Statement

The authors declare that the research was conducted in the absence of any commercial or financial relationships that could be construed as a potential conflict of interest.

## Footnotes

^1^http://www.picuscientist.org/pprep
